# Effect of Elevated CO_2_, O_3_, and UV Radiation on Soils

**DOI:** 10.1155/2014/730149

**Published:** 2014-02-06

**Authors:** Pavel Formánek, Klement Rejšek, Valerie Vranová

**Affiliations:** Department of Geology and Soil Science, Faculty of Forestry and Wood Technology, Mendel University in Brno, Zemědělská 3, 613 00 Brno, Czech Republic

## Abstract

In this work, we have attempted to review the current knowledge on the impact of elevated CO_2_, O_3_, and UV on soils. Elevated CO_2_ increases labile and stabile soil C pool as well as efficiency of organic pollutants rhizoremediation and phytoextraction of heavy metals. Conversely, both elevated O_3_ and UV radiation decrease inputs of assimilates to the rhizosphere being accompanied by inhibitory effects on decomposition processes, rhizoremediation, and heavy metals phytoextraction efficiency. Contrary to elevated CO_2_, O_3_, or UV-B decreases soil microbial biomass, metabolisable C, and soil N_*t*_ content leading to higher C/N of soil organic matter. Elevated UV-B radiation shifts soil microbial community and decreases populations of soil meso- and macrofauna via direct effect rather than by induced changes of litter quality and root exudation as in case of elevated CO_2_ or O_3_. CO_2_ enrichment or increased UV-B is hypothesised to stimulate or inhibit both plant and microbial competitiveness for soluble soil N, respectively, whereas O_3_ favours only microbial competitive efficiency. Understanding the consequences of elevated CO_2_, O_3_, and UV radiation for soils, especially those related to fertility, phytotoxins inputs, elements cycling, plant-microbe interactions, and decontamination of polluted sites, presents a knowledge gap for future research.

## 1. Introduction

Carbon dioxide, ozone, and ultraviolet radiation are individual climate change factors that have direct biological effects on plant coverage. Elevated CO_2_ causes up- and downregulation of genes of primary plant metabolism and N_2_ fixation; elevated O_3_ significantly diminishes the carbon sink of soil-plant systems [1, 2]. Both rising CO_2_ and surface O_3_ impact upon plant growth, response of crops to pests and herbivores, and the ability of plants to support decontamination of polluted sites [1]. Decrease in stratospheric O_3_ is accompanied by increasing UV radiation of which most attention has been given to UV-B. Elevated UV-B reduces crop yields and tree biomass, plant respiration potential, gas exchange, leaf area, and water-use efficiency and increases the content of amino acids, hormones, and flavonoids [3].

While replacement of current solvents by oxygenates decreases O_3_ pollution [4], forest fires increase O_3_ concentrations in some countries [5]. Calfapietra et al. [6] also reported formation of O_3_ from volatile organic compounds (especially isoprenoids) released from vegetation, which react in the atmosphere with NO_*x*_ to produce O_3_ under UV radiation. Many reviews focused on the effects of elevated CO_2_, O_3_, and UV-B radiation on plant biomass, ecosystems, and human health (e.g., [1, 7]). Nevertheless, only little work has focused upon understanding the consequences for soils. In this paper, we present a review of the current knowledge on the impact of elevated CO_2_, O_3_, and UV on soils and identify new hypotheses for future research.

## 2. Effect of Elevated UV Radiation on Soils

### 2.1. Direct Effect of UV Radiation on Soil Microorganisms

Pigment content, cell oxygen yield, growth, C assimilation, and PSII of cyanobacteria change with increasing UV-B [8]; besides, UV-B also induces synthesis of mycosporine-like amino acids [9]. Soil surface bacteria are more resistant to UV than subsurface bacteria [4]. Nonmotile Gram-positive bacteria isolated from Antarctic soils are tolerant to UV radiation due to synthesis of protective melanins [10]. Also, compost-born thermophilic methanogenic Archaea were proved to be resistant to UV-B, probably due to their attachment to compost material acting as an effective carrier [11]. Growth of lichens is not affected by UV-B due to increased phenolics content [12].

Direct effects of UV on soils occur through a shift of the fungal community with an increase in competitive abilities of darkly pigmented fungi [13]. Only some of the soil and phylloplane fungal species are sensitive to UV-B [14]. For example, the entomopathogenic fungus *Tolypocladium* sp. is UV-B tolerant [15]. Peatland amoebae are more abundant in ambient than reduced UV-B and diversity of some species increases under ambient UV-B [16].

### 2.2. Direct Effect of UV Radiation on Soil Meso- and Macrofauna

UV-B pretreatment decreases rotifers, nematodes and mites population size and increases generation time in soils polluted with heavy metals due to reproductive defects; nevertheless, it protects *Caenorhabditis elegans* from disturbed locomotion [16, 17]. Experiments showed that a large increase in nematode density in Antarctic soils (especially microbivorous genus *Plectus*) resulted from blocking UV with a UV-absorbing perspex cloth [18]. No effect of UV-B on the mass of earthworms feeding on litter was found, and some of the species benefited from UV-B [19]. Low mortality of spider mites due to UV-A, UV-B, and UV-C was reported by Suzuki et al. [20], while inactivation of *Ascaris* eggs was significant only in water [21].

### 2.3. Release/Degradation of Soil Pollutants by UV Radiation

Elevated UV-B (but not UV-A) directly reduces soil-associated Hg through significant increase of Hg emissions from forest soils [22]. UV-B is also known to increase degradation of pollutants (phenylurea herbicides, p,p′-DDT, 2,4-dichlorophenoxyacetic acid, biphenol, Z or PAHs), while PAHs degradation on soil surfaces, in the presence of nanometer anatase TiO_2_, follows pseudo-first-order kinetics [23]. UV photolysis has been suggested as a suitable treatment for extracts of PAHs contaminated soils, where up to 83% removal was achieved [24].

### 2.4. Measured Effects of UV-B Radiation on Soils

**Figure 1:**
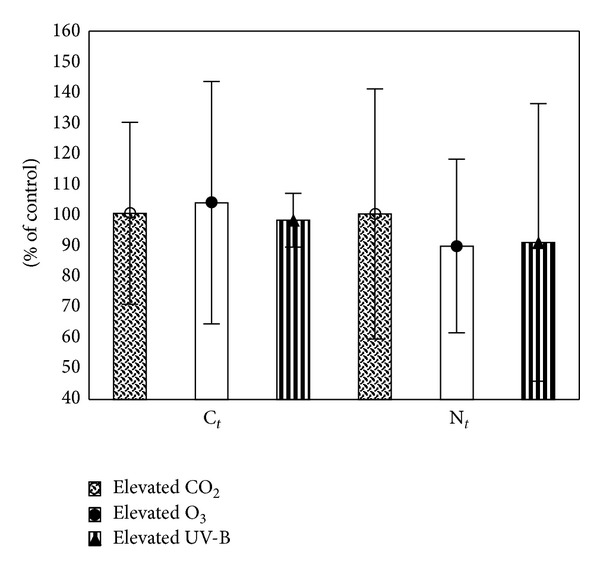
Effect of elevated CO_2_, O_3_, and UV-B on C_*t*_ and N_*t*_ (recalculated from [30–33]). The values are expressed in % of control = 100% which represent ambient CO_2_, O_3_, or UV-B (mean ± SD).

**Figure 2:**
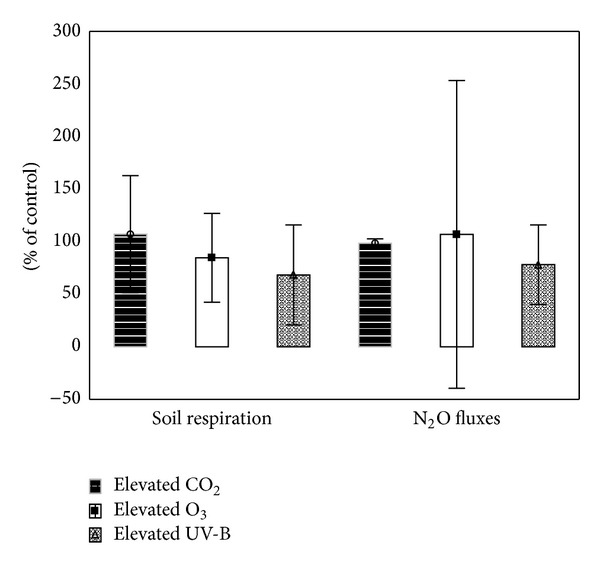
Effect of elevated CO_2_, O_3_, and UV-B on soil respiration and N_2_O fluxes (recalculated from [27, 34–40]). The values are expressed in % of control = 100% which represent ambient CO_2_, O_3_, or UV-B (mean ± SD).

**Figure 3:**
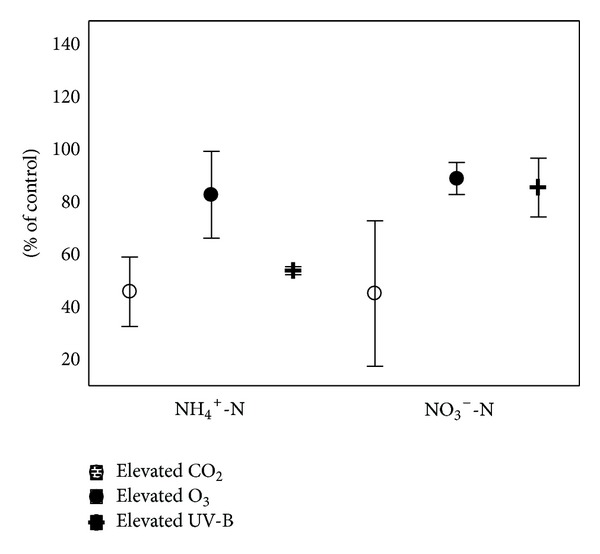
Effect of elevated CO_2_, O_3_, and UV-B on NH_4_^+^-N and NO_3_^−^-N content in soil (recalculated from [16, 27, 33, 41]). The values are expressed in % of control = 100% which represent ambient CO_2_, O_3_, or UV-B (mean ± SD).

Elevated UV-B does not substantially influence initial chemical composition of leaf litter [25] and has only little effect on total carbon (C_*t*_) and nitrogen (N_*t*_) in soils; on average they decrease by 2 and 9%, respectively (Figure 1). On the other hand, elevated UV-B decreases NH_4_^+^-N and NO_3_^−^-N by 46 and 14%, respectively (Figure 3), and reduced UV-B (compared to ambient value) decreases dissolved organic carbon (DOC) and phosphorus content in 0–10 mm of peatland in course of vegetation [16]. Pretreatment of air-dried litter with UV followed by rewetting did not change decomposition rate [26], whereas some researchers found inhibitory effect of UV-B on soil organic matter (SOM) decomposition (reduced by 32% on average) with no effect on Q_10_ (Figure 2). Lower effect later in the season occurs due to increasing crop coverage reducing soil sterilization [27]. Also, N_2_O fluxes in soils are reduced by elevated UV-B by ca. 22% with no change of diurnal variation patterns (Figure 2). Elevated UV-B equivalent to 15% O_3_ depletion decreases N_2_ fixation in tropical leguminous crops due to reduced photosynthesis and nodulation including nitrogenase activity; nevertheless, the molecular basis of this phenomenon is not known yet [28]. Altered gene activity due to elevated UV-B was found to enhance rice allelopathic potential (inhibition and stress of neighbouring plants especially at high density in native environment) including autotoxicity via phytotoxins of root exudates and leaf leachates [29] of which identification presents a knowledge gap for future research.

### 2.5. Hypotheses on Indirect Effects of UV Radiation on Soils

Plant coverage ameliorates the impact of elevated UV-B on soil microorganisms; nevertheless, indirect effects via altered quality and reduced quantity of plant biomass are hypothesised to inhibit SOM decomposition and heavy metals bioremediation. These include especially accumulation of phenolics, salicylic acid, tannins, cinnamic acid, and flavonoids [3]. Phenolics are involved in stabilization of aggregates and some of them (e.g., gallic acid) decrease cation exchange capacity (CEC) of soils; on the other hand, hydrolysable tannins (e.g., *β*-1,2,3,4,6-penta-O-galloyl-D-glucose) increase the CEC [42]. Phenolics and flavonoids are inhibitors of decomposition processes including enzymatic activities (sulphatase, phosphatase, *β*-glucosidase, xylosidase, chitinase, and dehydrogenase) and are also involved in stabilization of xenobiotics and Fe complexation representing a potential constraint in wetland-based acid mine drainage bioremediation, due to low Fe availability (e.g., [43, 44]). Phenolics are also known to support growth of PCB-degrading bacteria [45].

## 3. Effect of Elevated CO_**2**_ on Soils 

### 3.1. Alteration of Soil Properties due to Elevated CO_2_

Elevated CO_2_ delays soil water depletion due to partial plant stomatal closure and alters solarization efficiency and heat fluxes [46, 47]. Furthermore, dilution of plant biomass by carbohydrates and increased plant-derived C inputs including rhizodeposition are hypothesised to increase C_*t*_ with no effect on N_*t*_ and reduction of mineral nitrogen content in soils [48]. Nevertheless, recalculation of data from a range of studies showed negligible effect of elevated CO_2_ on soil C_*t*_ and N_*t*_ (Figure 1). Effect of elevated CO_2_ on soil C_*t*_ and N_*t*_ is ecosystem- and type of plants-dependent being increased only in sweetgum or cotton plantations, deserts, *Agrostis capillaris* cover, or seminatural grasslands (Table 1). It may also be affected by the initial soil properties, the type of experiment (laboratory *versus* field), occurrence of N_2_-fixing species, and the plant C allocation pattern being affected by plant genotypic variation [34]. For example, the effect of elevated CO_2_ on soils may be diminished in base-rich sites [49]. Contrary to cultivated plants, wild genotypes allocate more C into roots resulting in greater rhizodeposition under elevated CO_2_ [34, 48].

Proportion of labile to recalcitrant C fraction changes in response to elevated CO_2_ via increased transfer of C into slow-decay C pool and reduces decay of old C; some works describe rhizodeposition-induced decomposition of stable soil C; quality and quantity of the labile C are altered by increased plant litter and root exudation [34, 35]. Elevated CO_2_ increases decomposition of metabolisable C only in topsoils with opposite effect in subsoils and no effect on amides degradation [34]. Root biomass and volume of rhizospheric soil including mycorrhizal symbiosis of trees in boreal and temperate zones increase due to elevated CO_2_ [34, 48]. In some ecosystems (peatlands), growth of root biomass of low decomposability is induced by elevated CO_2_, and in some cases, decomposition of fine roots is faster [50]. Concentrations of both mineral nitrogen forms (NH_4_^+^-N and NO_3_^−^-N) in soils are significantly reduced (by >50% on average) by elevated CO_2_ (Figure 3), probably due to N dilution in foliage and increased plant-microbes competition for N sources [48]. Furthermore, elevated CO_2_ may alter the chemistry of groundwater (Ca^2+^, trace metals and other types of cations and anions) [48] and its effect on bulk density or pH of soil is low (Table 1).

### 3.2. Elevated CO_2_ versus Soil Microbial Community and Activity of Enzymes

Elevated CO_2_ (including transient elevation) changes the structure and physiology of soil microbial community in favour of bacteria due to lower soil nitrogen inputs which are accompanied by reduction of the abundance of taxonomic units within the *Firmicutes* as well as the populations of Gram-positive bacteria in rhizosphere soils [54, 55]. Allocation of C to soil microorganisms usually depends on the type of ecosystem [34, 56] and is often accompanied by increased C_MIC_/C_*t*_ ratio. An increase of soil microbial N (N_MIC_) as a posttreatment response to elevated CO_2_ in N-limited ecosystems was found probably due to lower nutrient (nitrogen) competition between microorganisms and plants [57]. Soil respiration increased by 7% on average due to elevated CO_2_ (Figure 2) compared to ambient control without change of substrate use efficiency [35] and N_2_O fluxes were only slightly changed (1.5% decrease) (Figure 2).

Numbers of archaeal and bacterial 16S rRNA and genes encoding key enzymes of ammonia-oxidation (*amoA*), denitrification (*nirK*, *nirS*, and *nosZ*), and genes of nitrate-reducing bacteria (*narG*, *napA*) are increased or reduced (or not affected) in the rhizosphere by elevated CO_2_ depending on inputs of fertilizers (N), soil depth and moisture, type of plant metabolism (C_3_ versus C_4_), time of sampling during growing season (start, peak, or senescence), and sampling year [58, 59]. Reduced crenarchaeal sequences and altered abundance of 16S rDNA as well as *amoA* genes of archeal community or enhanced fungal cellulolytic community gene *cbhl* fragment richness due to elevated CO_2_ were found in the rhizosphere of C_3_ but not C_4_ plants [58]. Abundance of red-like *cbbL* genes of CO_2_-fixing bacteria is reduced and diversity of soil purple phototrophic bacteria increased in conditions of elevated CO_2_ [58, 59]. N fertilization increases abundance of bacterial *amoA* gene only under ambient CO_2_ whereas an opposite effect occurs for thaumarchaeal *amoA* gene [58, 59].

Elevated CO_2_ induces an increase of soil enzymatic activities (protease, xylanase, invertase, phenol oxidase, alkaline phosphatase, and arylsulphatase) in the main rooting zone due to enzyme regulation (synthesis and activity) via enlarged pool of easily available substrates rather than by shifts in microbial abundance [57]. Stimulation of plant root-derived enzymatic activities due to enhanced photosynthesis is also hypothesised under elevated CO_2_ [43].

### 3.3. Effect of Elevated CO_2_ on Soil Meso- and Macrofauna

Elevated CO_2_ suppresses the role of fauna in litter decomposition due to its dilution by carbohydrates and the effect is ecosystem-dependent being significant especially in tropical forests [60, 61]. Elevated CO_2_ modifies the pattern (abundance and diversity) of nematode communities (especially groups of omnivores, saprophagous feeders, and predators), earthworms and enchytraeids, oribatid mites, microarthropods, collembolans, and omnivorous insects as well as the proportion of edaphic groups via changes of plant biomass quality and moisture [61]. Elevated CO_2_ has generally been found to have negative impacts on the performance of insect herbivores whose larvae reach smaller size when feeding on elevated CO_2_-grown plants [61]. The hypothesised negative impact of elevated CO_2_ on omnivorous bugs via lowering the quality of plants and prey was not proved; on the contrary, the predators may benefit from elevated CO_2_ through increased vulnerability of their prey [60, 61].

## 4. Effect of Ozone on Soils

### 4.1. Alteration of Soil Properties due to Elevated O_3_

Effect of elevated O_3_ on soils is poorly understood. O_3_ deposition to soil has been expressed by parameters such as aerodynamic resistance (*R*_*a*_), quasilaminar boundary layer resistance (*R*_bO3_), and soil resistance (*R*_soil_) being a function of soil water content with daily variations [62]. Ozonation of humic acids or their components (p-hydroxybenzaldehyde, vanillin, syringaldehyde, vanillic acid, and di-n-butylphthalate) leads to formation of mutagenic compounds and O_3_ also induces amino acid racemization [63].

Contrary to elevated UV-B or CO_2_ which cause N dilution and increased phenolics in plant biomass, elevated O_3_ modifies plant biomass via decrease in both N and phenolics [36, 64]. Elevated O_3_ increases C_*t*_ and reduces N_*t*_ by 4 and 10%, respectively (Figure 1); NH_4_^+^-N and NO_3_^−^-N are also reduced by 17 and 10%, respectively (Figure 3), including humic acids fraction, C_MIC_, and pH of soils in different ecosystems (black cherry, milkweed, spring wheat, and beech) [19]. Soil respiration is decreased (by 15% on average) (Figure 2) under O_3_ enrichment; the same was found in case of methane emissions from soils of different ecosystems (e.g., temperate lowland peat bogsor rice soils) which are reduced by about 25% [65]. On the other hand, N_2_O fluxes are enhanced (by 7% on average) under O_3_ enrichment (Figure 2) as it reacts with N_2_O emitted from fertilized soils [37]. Elevated O_3_ increases Ca^2+^, Mg^2+^, and Mn^2+^ in soil solution and stimulates export of NO_3_^−^ from forest sites [19].

### 4.2. Alteration of Soil Microbial Communities and Fauna by Elevated O_3_

Elevated O_3_ alters soil microflora structure and physiology with a negative impact on numbers of bacteria and fungi, glutathione content of protozoa, and His^+^ reversion of some bacteria [30]. Especially the numbers of functional microbial genes are lower under O_3_ treatment in dependence on plant coverage development and N fertilizers inputs with no effect on *amoA* and *nosZ* genes abundance [66]. Increased terpene inputs (especially *α*- and *β*-pinene or 3-carene) as a consequence of elevated O_3_ are hypothesised to alter soil microbial community, especially via stimulation of bacteria and inhibition of fungi [67]. Roots of forest trees are also a significant source of monoterpenes in soil and over 75% of ectomycorrhizal fungi or 25% of isolated saprotrophic fungi were inhibited by one of the monoterpenes, affecting the structure of the fungal community [67]. On the other hand, monoterpenes supplied to soil increase degradation of polychlorinated biphenyls (PCB), even without increasing the bacterial biomass [68]. Elevated O_3_ strongly decreases the abundance rather than genera richness of soil collembolans compared with ambient atmospheric O_3_, probably due to decreased allocation of carbohydrates to roots. However, in the Bt cotton fields, elevated O_3_ did not significantly affect the abundance or diversity of soil collembola suggesting that Bt cotton can buffer the effect of elevated O_3_ on soil collembolans via root-derived ways [69]. Contrary to elevated CO_2_ and UV radiation, elevated O_3_ is hypothesised not to increase phytotoxicity of soils through inputs of plant phenolics [36]; nevertheless, a direct effect via racemization of low-molecular-weight organic compounds (amino acids) is hypothesised [63].

## 5. Hypotheses on the Effect of Elevated CO_**2**_, O_**3**_ and UV Radiation on Plant-Microbe Competition for N Sources

Plant communities, especially at low productivity sites with acid soils, are more adapted to organic N uptake; nevertheless, higher proportion of both soil organic as well as mineral N is captured by microorganisms rather than by plants [48]. Management practices including grasslands mowing or grazing or forest stands thinning are thought to reduce plant-microbe competition for N sources, since N-cycling and mineralization rates are increased and are accompanied by lower organic N availability and no effect on kinetics of organic N uptake by microorganisms [48].

O_3_ enrichment shifts the N-balance in favour of plants over soil microorganisms, being significant especially in ecosystems with low productivity (grasslands, mountain forests, and tundra communities) where organic N forms the dominant pool [48]. Alterations in plant-microbe competition for N sources may be facilitated by natural fungicides (phenolics) which is hypothesised to be significant in case of elevated CO_2_ and UV, but not O_3_ [64]. In this case, dominance of the bacterial fraction of the soil microbial community favours microbial competitiveness over plant roots; however, this advantage may be eliminated by fertilization [48]. Also, it is hypothesised that the maximum N acquisition by plants is regulated by intermediate concentrations of phenolics [64]. Elevated O_3_ reduces ascorbic acid in plant biomass; its degradation in soils may act to produce an effective sporocide, which plays a role in mitigation of salinity effects on plant growth [70]. Competition between microbes and plants for N sources is regulated by rhizodeposition in terms of exudation rates and qualitative composition of the exudates, both being altered by elevated UV-B, CO_2_, and O_3_ [48]. Nitrifiers are strong competitors for NH_4_^+^ on fertile sites where competition may be reduced due to increased tannins under elevated UV-B or CO_2_ [48].

## 6. Conclusions

Overall, study of the consequences of elevated CO_2_, O_3_, and UV radiation for soils is significant due to increasing CO_2_ concentrations worldwide and also because there is clear evidence that stratospheric O_3_ is being depleted which causes increased ground UV radiation. On the other hand, change of air quality due to emissions of hydrocarbons and exhaust gases leads to increasing tropospheric O_3_ production. Contrary to elevated CO_2_, O_3_, or UV-B decreases C_MIC_, metabolisable C, and soil N_*t*_ content leading to higher C/N of soil organic matter. Mechanism of the CO_2_ or O_3_ enrichment effects on soils including elevated UV-B radiation differs considerably. Elevated O_3_ or UV-B decreases inputs of assimilates to the rhizosphere and has an inhibitory effect on decomposition processes and rhizoremediation of organic pollutants. UV-B shifts soil microbial community and decreases populations of soil meso- and macrofauna directly rather than via induced changes of litter quality and root exudation as in case of elevated CO_2_ or O_3_. Worldwide increasing CO_2_ concentrations stimulate rhizoremediation of organic pollutants due to higher root biomass and volume of rhizospheric soil as well as phytoextraction of heavy metals (contrary to elevated O_3_ or UV-B) as a result of increased mycorrhizal colonization and plant biomass. Enhanced C inputs and root mycorrhizal colonization as a consequence of elevated CO_2_ are hypothesised to stimulate both microbial and plant N acquisition. UV-B is hypothesised to reduce both plant and microbial competitiveness for soluble soil N whereas O_3_ enrichment favours microbial competitive efficiency. Since the effects of elevated CO_2_, O_3_, and UV radiation on soils are only little understood, it is essential to conduct further studies to understand their consequences for soil fertility, elements cycling, and decontamination of polluted sites.

## Figures and Tables

**Table 1:** Effect of elevated CO_2_ on soils according to type of ecosystem.

Type of ecosystem	Soil properties	References
Deciduous and coniferous forests	Increased or decreased C/N	[42]
Poplar cultivation	No effect on C_ox_, N_*t*_ and bulk density, increased humification, increased or reduced export of DOC, increased leaching of refractory C	[35]
Rice-wheat rotation	Decreased available N by up to 50% and available P by 30%	[50]
Oak ecosystem	No effect on total organic carbon (TOC), 30% reduced slow-degradable C, 41% increased C_MIC_, no effect on pH	[50]
C_4_ plant communities	Decreased N mineralization	[36]
*Agrostis capillaris *	Increased C_ox_ and decreased C/N	[30]
*Lathyrus pratensis *	Decreased C_ox_ and increased C/N, decreased soil bacteria and mycorrhizal fungi	[30]
*Plantago lanceolata *	Increased net N mineralization	[31]
Different ecosystems	Decrease in relative abundance of Acidobacteria Group 1 bacteria, increased fluxes of CO_2_, NH_3_, N_2_O, and CH_4_ with induction of CH_4_ oxidation	[39]
Pine ecosystem	No effect on soil properties	[39]
Sweetgum plantation	Increased C_ox_, no effect on soil microbial community, enzymatic activity, potential N mineralization and nitrification	[33]
Seminatural grasslands	Increased C_ox_ and N_*t*_	[51]
Cotton plantation	Increased C_ox_ only under wet moisture regime, no effect on N_*t*_	[52]
Deserts	Increased C_ox_ and N_*t*_ under some types of coverage, low effect on soil pH and bulk density, no effect on arbuscular mycorrhizas; no effect on fluxes of CO_2_, NH_3_, N_2_O, and CH_4_	[53]
